# Wallerian Degeneration and Nerve Regeneration—A Review of Cellular and Molecular Events

**DOI:** 10.3390/ijms27146368

**Published:** 2026-07-17

**Authors:** Petr Dubový

**Affiliations:** Cellular and Molecular Neurobiology Group, Department of Anatomy, Faculty of Medicine, Masaryk University, CZ-62500 Brno, Czech Republic; pdubovy@med.muni.cz

**Keywords:** traumatic nerve injury, extrinsic conditions, retrograde signaling, neuronal regeneration program, axon regeneration

## Abstract

Wallerian degeneration (WD), which occurs distal to peripheral nerve injury, is a tightly regulated process. Axonal degeneration during the onset of WD represents a self-destructive process that begins with an early phase and progresses to an execution phase characterized by fragmentation of axons and myelin sheaths. Efficient clearance of axonal and myelin debris, together with the reprogramming of Schwann cells and macrophages into a repair phenotype, constitutes a critical extrinsic prerequisite for successful axonal regeneration. In parallel, signaling molecules produced during WD trigger intrinsic responses in injured neurons that are essential for neuronal survival and the initiation of the regenerative program. This review provides an overview of the key events involved in WD, which are often studied separately, with the aim of elucidating their interrelationships and their impact on nerve regeneration. Such an integrated overview may aid in identifying molecular targets for the development of novel therapeutic strategies to enhance axonal regeneration.

## 1. Introduction

Wallerian degeneration (WD) in a peripheral nerve occurs when axonal integrity is compromised by traumatic, toxic, ischemic, or metabolic events [1]. Most fundamental insights into the cellular and molecular processes of WD and subsequent axon regeneration have been obtained from experimental injuries to peripheral nerves and axons under in vivo and in vitro conditions, respectively. A type of peripheral nerve injury, where axons are disconnected from cell bodies and axonal transport is interrupted, triggers a cascade of cellular and molecular changes that are considered part of an innate immune reaction or neuroinflammation [2,3].

The key cellular events of WD include axon and myelin fragmentation [1,4]; dedifferentiation of Schwann cells and their transformation into a repair phenotype [5]; breakdown of the blood–nerve barrier [6,7]; and recruitment of immune cells, including neutrophils and macrophages [8,9]. Both Schwann cells and macrophages contribute to cleaning axonal and myelin debris, thereby creating extrinsic conditions that promote axon regeneration.

The proximal stump of disrupted peripheral axons can regenerate and reinnervate target structures if critical conditions are met. These include the survival of neuronal cell bodies, the activation of the intrinsic regeneration program, and the creation of a permissive cellular and extracellular environment. Key molecular signals generated during WD induce a repair phenotype in Schwann cells and activate the intrinsic neuronal regeneration program [10,11,12]. In addition, dynamic alterations in the components of the endoneurial extracellular matrix (ECM) significantly contribute to the formation of a permissive milieu for axon regrowth [13,14]. These endoneurial changes, together with the maintenance of Schwann cell specificity for axon type, play a crucial role in guiding regenerating axons to their appropriate peripheral targets [15,16,17].

Recent advances in our understanding of WD have suggested its dual role as both a degenerative process and a crucial contributor to the cellular and molecular conditions required for axon regeneration. New findings have refined, expanded, and in some cases revised previous models of the cellular and molecular mechanisms underlying WD. Furthermore, this review critically evaluates current knowledge of the cellular and molecular mechanisms involved in WD, particularly with regard to the search for targets that may facilitate the development of novel therapeutic strategies to improve axon regeneration.

## 2. Axon Degeneration

Although the time course of axonal degeneration differs between in vitro and in vivo models, it is generally divided into early (latent) and late (execution) phases. Since axonal degeneration occurs at the onset of WD, the terms “early” and “late” phases here are used to refer to indistinct and visible structural changes in injured axons, respectively. In the early phase, damaged axons appear morphologically intact, while the late phase is characterized by rapid fragmentation of axons and their myelin sheaths distal to the injury site. However, the transition between these phases is gradual, as many processes progress continuously from the early to late stages.

### 2.1. Early Phase of Axonal Degeneration

While the structural disintegration of damaged axons distal to a nerve injury may not be immediately apparent in the early phase, axon degeneration is initiated by an intrinsic self-destruction program. This process is orchestrated by the influx of ions, the activation and inhibition of specific proteins, and the subsequent disruption of axonal transport [18]. The early phase of axonal injury can last approximately 1–2 days under in vivo conditions [19].

The endoplasmic reticulum and mitochondria are the primary regulators of axoplasmic calcium levels under normal conditions [20]. An increase in calcium concentration following axonal injury is one of the earliest triggers of subsequent cellular and molecular changes. Immediately after axonal injury, a rapid influx of extracellular calcium occurs at the injury site due to disruption of the axonal membrane, reversal of the sodium/calcium exchange pump, and calcium release from the endoplasmic reticulum. This calcium wave propagates along injured axons [12,21,22]. Elevated intra-axonal calcium levels lead to mitochondrial calcium overload, thereby triggering mitochondrial dysfunction characterized by a decline in ATP production and increased oxidative stress, with enhanced production of reactive oxygen species (ROS). This is followed by activation of the mitochondrial permeability transition pores (mPTPs) and the release of ATP and other mitochondrial damage-associated molecular patterns (mtDAMPs) [19,23,24]. Thus, calcium influx into mitochondria and the activation of mPTPs represent additional parallel events during early axonal degeneration [25,26], which are linked to subsequent cellular and molecular processes in WD.

Swelling is an early visible structural response of axonal mitochondria to axotomy. In vitro experiments revealed minimal mitochondrial swelling at 6 h, with an almost twofold increase in mitochondrial diameter observed at 12 h. A comparison of these mitochondrial changes with the visibly granular disintegration of axons under in vitro conditions suggested that mitochondrial swelling precedes the loss of axonal integrity [25]. These results were confirmed by in vivo experiments [27].

Mitochondria are among the most abundant organelles in axons, although their distribution along the axon is not uniform. This may contribute to some of the inconsistent results observed in experimental studies. The primary functions of mitochondria are to generate ATP and buffer intra-axonal calcium levels; thus, their dense distribution along peripheral axons is commonly associated with the juxtaparanode and internode axonal segments of Ranvier’s nodes, Schmidt–Lanterman clefts, and axon terminals [28,29,30]. It is therefore not surprising that mitochondria are among the earliest and most sensitive responders to axon injury, both at the molecular and structural levels [19]. Monitoring mitochondrial dynamics distal to axon injury is essential, as mitochondria play key roles in both the early and late stages of axonal degeneration.

With early mitochondrial changes, other important molecular processes associated with the metabolic balance of nicotinamide adenine dinucleotide (NAD^+^) and its precursors become disrupted. Nicotinamide mononucleotide adenylyltransferases (NMNATs) catalyze the synthesis of NAD^+^, a vital cofactor for neuronal energy metabolism, including mitochondrial functions such as ATP production via the respiratory chain. NMNAT3, an mitochondrial isoform of NMNAT, plays an essential role in NAD^+^ synthesis and is involved in the regulation of ROS and oxidative stress [31]. Under physiological conditions, mitochondria regulate ROS generation; however, increased axonal ROS triggered by imbalances in the mitochondrial electron transport chain is another factor contributing to axonal injury. This suggests that mitochondrial NMNAT3 could be a key factor in axonal protection during WD, although further research is needed to clarify its specific role [32].

In contrast, NMNAT2, an isoform located in the Golgi apparatus and Golgi-derived vesicles in the neuronal soma, is required for the fast axonal transport of vesicular cargo [33]. Under physiological conditions, NMNAT2 continuously converts nicotinamide mononucleotide (NMN) into nicotinamide adenine dinucleotide (NAD^+^), thereby maintaining NAD^+^ levels and a low NMN/NAD^+^ ratio. Simultaneously, the sterile-α and Toll/interleukin-1 receptor (TIR) motif-containing 1 protein (SARM1) remains inactive. This inactivity is mediated by the binding of NAD^+^ to the autoinhibitory armadillo/heat repeat motif (ARM) domain, which prevents activation of nicotinamide adenine dinucleotide nucleosidase (NADase) in SARM1 [34]. This makes NMNAT2 a critical enzyme for maintaining axonal integrity. Disruption of its axonal transport, or its depletion from the injured axonal segment, is associated with further molecular changes during WD [1,35,36,37]. Reduced NMNAT2 levels lead to a decrease in NAD^+^, resulting in the accumulation of NMN and an increased NMN/NAD^+^ ratio within injured axons. NMN, in turn, binds to the ARM domain of SARM1, thereby activating its NADase, which further depletes NAD^+^ [38]. In contrast to the protective role of NMNAT2, SARM1 is required for axon degeneration [18,39,40]. SARM1 is involved not only in axon degeneration following traumatic nerve injury but also in chemotherapy- or diabetes-induced peripheral neuropathies [40,41,42]. Therefore, inhibition of the SARM1 pathway using small-molecule or allosteric cysteine inhibitors has emerged as a promising therapeutic strategy for reducing axon degeneration in clinically relevant disorders [43,44,45,46]. The neuroprotective potential of SARM1 inhibition has been demonstrated in preclinical models of nerve injury and disease [47]. Furthermore, its ability to restore an intermediate pool of damaged axons to a healthy state may have significant therapeutic implications for the treatment of Charcot–Marie–Tooth type 2A, an approach that is currently in preclinical testing [48,49]. In addition, experimental evidence indicates that SARM1 inhibition may delay WD following nerve transection and promote earlier recovery of motor function [50]. However, the potential clinical utility of SARM1 inhibition requires further investigation, as sub-inhibitory concentrations of SARM1 inhibitors have been shown to paradoxically activate SARM1 and accelerate neurite degeneration in neuronal assays [51].

SARM1 is a multidomain protein, with each domain having distinct functions [52,53], suggesting that SARM1 may mediate multiple injury-induced responses beyond its established role in axon degeneration. For example, traumatic axonal injury activates an intrinsic neuronal immune capacity that is spatially and temporally separated from injury-induced axon degeneration. This neuronal immune response is regulated by the TIR-domain adaptor protein Sarm1/Myd88-5 via its downstream JNK/c-JUN signaling pathway [54]. However, direct evidence that SARM1 supports the neuronal regeneration program has not yet been provided.

Interestingly, genetic elimination of *Sarm1* delays macrophage recruitment and immunophenotypic switching [55], as well as the appearance of repair Schwann cells in the injured nerve [56]. These findings suggest that SARM1, a key regulator of programmed axon degeneration, also plays a critical role in the early response of non-neuronal cells to axonal injury, including macrophage recruitment and Schwann cell reprogramming (see below).

NMNAT2 has a short half-life, which is regulated by increased mitogen-activated protein kinases (MAPKs), particularly dual-leucine zipper kinase 1 (DLK-1) and leucine zipper-bearing kinase (LZK). DLK-1, also known as mitogen-activated protein kinase kinase kinase 12 (MAP3K12), promotes axonal self-destruction through the downstream activation of the c-JUN N-terminal kinase (JNK) pathway [57]. Accordingly, the DLK-1/JNK signaling axis plays a key role in initiating the axon degeneration program. Notably, beyond its role in promoting axonal disintegration during the early phase of WD, increased DLK-1 in response to axonal injury is also linked to retrograde JNK signaling, which is required for initiating the neuronal regeneration program (see Section 5).

Another direct target of axonal JNK is stathmin 2, also known as Superior Cervical Ganglion 10 (SCG10). SCG10 is co-transported with NMNAT2 from neuronal bodies into axons and is rapidly depleted by phosphorylation-dependent proteasomal degradation during the early phase of axon injury [18,58]. Moreover, the rapid depletion of SCG10 distal to the site of axonal injury, along with its upregulation in neuronal cell bodies and proximal axonal stumps, makes the SCG10 protein a suitable marker for visualizing regenerated sensory axons [59].

In summary, axotomy-induced early elevations of calcium levels and disruptions in axonal transport are associated with the rapid depletion of NMNAT2, an imbalance in the NMN/NAD^+^ ratio, mitochondrial dysfunction, and activation of SARM1, which collectively trigger the subsequent steps of axonal degeneration. In the context of the critical role of SARM1 in axon degeneration and current development of SARM1 inhibitors that attenuate peripheral neuropathies, it will be important to determine whether these inhibitors also impair axonal regeneration.

### 2.2. Late Phase of Axonal Injury

Despite some discrepancies arising from the use of different experimental models and measurement techniques in the study of calcium dynamics, two waves of axonal calcium elevation can be defined following axon injury. The first wave involves a rapid, injury-induced increase in calcium levels at the site of damage, followed by a second phase characterized by elevated cytosolic calcium along the entire length of the axon [20,22].

During the transition from the early to the late phase, axonal structural damage is initiated by SARM1, which is also associated with the second wave of calcium accumulation [60]. The execution phase of axonal degeneration is characterized by the activation of calcium-dependent proteases, such as calpains. The activity of these enzymes leads to the granular disintegration of the axonal cytoskeleton and other structures into small fragments, which are subsequently cleared by phagocytic cells [61,62,63].

**Figure 1:**
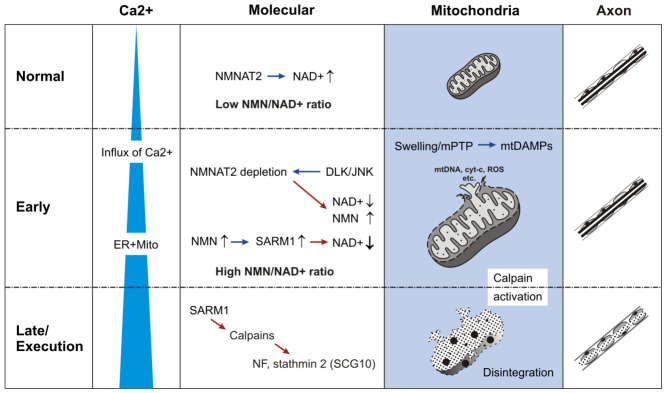
Diagram illustrating molecular interactions and key mitochondrial responses associated with Ca^2+^ influx, focusing on NMNAT2, NMN, SARM1, and DLK and their relationships during the early and late phases of axonal injury.

The molecular interactions associated with mitochondrial responses during the early and late phases of axonal injury are summarized in Figure 1.

## 3. Pro-Inflammatory Phase of Wallerian Degeneration

Wallerian degeneration creates a microenvironment distal to the site of nerve injury that supports axonal regrowth and regeneration. This process can be divided into pro-inflammatory and pro-regenerative phases, which comprise an overlapping sequence of molecular and cellular events occurring both at and distal to the nerve lesion. The pro-inflammatory phase of WD is initiated by the disintegration of axons and myelin sheaths into cellular debris, followed by its clearance. Schwann cells that have lost axonal contact begin actively phagocytosing the detached debris, proliferate, and dedifferentiate. They rapidly upregulate c-JUN and secrete pro-inflammatory cytokines and chemokines, including TNF-α, IL-1β, CCL2, and CSF1, thereby changing into an inflammatory phenotype. Pro-inflammatory Schwann cells recruit circulating neutrophils, monocytes, and macrophages at the site of nerve injury and along the distal nerve stump [2,5,64,65].

### 3.1. Schwann Cells as First Responders to Axonal Injury

Due to intimate contact with axons, Schwann cells are expected to respond rapidly to pathological changes following axonal injury. This early reaction is initiated at the sites of direct axon–glial interaction [66]. In the case of myelinating Schwann cells, early signaling from injured axons occurs at peripheral terminal segments, the nodes of Ranvier, and the Schmidt–Lanterman clefts of sensory and motor axons.

In response to axon degeneration, Schwann cells undergo a well-orchestrated phenotypic transition characterized by proliferation and functional reprogramming that support axon regeneration. These complex processes are driven by dynamic transcriptional regulations involving coordinated downregulation and upregulation of numerous genes, leading to extensive changes in protein expression.

Recently, the spatiotemporal regulation of Schwann cell responses during WD has been investigated in detail using longitudinal single-cell transcriptomic analysis of rat sciatic nerve injury at multiple time points. Following nerve injury, Schwann cells under-went gradual phenotypic transitions through five distinct cell clusters characterized by specific marker genes, progressing from pro-inflammatory states to repair Schwann cells and then to a remyelinating subpopulation [67]. Spatial transcriptomics has led to further advances in the knowledge of Schwann cell transition during WD [68,69,70], indicating not only that Schwann cell transcriptomic stages are controlled by both positive and negative transcription regulators [71] but also that their preferential location is at the borders of nerve lesions [68,69,71,72]. Here, Schwann cells dynamically interact with macrophages, which influences the transition of fibroblasts into myofibroblasts [73], and then they migrate into fibroblast-rich spaces [72].

A more precise understanding of the functional roles of individual Schwann cell subpopulations, as well as the significance of the gene expression changes identified by longitudinal scRNA-seq studies, requires further experimental investigation. The following sections review the mechanisms that govern Schwann cell responses during WD and their transition to the repair Schwann cell phenotype, a critical cellular process underlying peripheral nerve regeneration.

### 3.2. Removal of Axonal and Myelin Debris Distal to Injured Nerve

The myelin sheath is a multilamellar structure derived from the spiral wrapping of the plasma membrane of myelin-forming Schwann cells around the axons. The production of the myelin sheath during peripheral nerve maturation is regulated by axonal signaling, such as neuregulin 1 (NRG1) type III activity and cell adhesion molecules [11,74]. The myelin contains molecules such as myelin basic protein (MBP) and other molecular components that create a non-permissive environment for axonal growth and inhibit or at least slow down the growth of regenerating axons [64,75,76]. Although axon regeneration has been observed within nerve segments containing myelin, efficient degradation of myelin debris remains essential for subsequent axonal regeneration and successful functional recovery after nerve injury [77,78].

**Figure 2:**
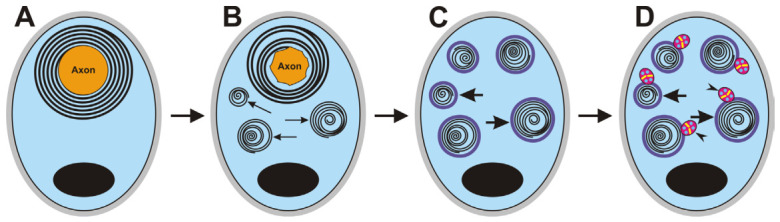
Schwann cells degrade the myelin sheath after axonal injury via a novel form of selective autophagy, known as myelinophagy. (**A**) Schwann cell and myelin sheath under normal conditions. (**B**) After axonal injury, the myelin sheath breaks down into intracellular myelin debris (arrows). (**C**) Autophagosomes (thicker arrows) develop from myelin fragments. (**D**) Autophagosomes (thicker arrows) fuse with lysosomes (arrowheads) to degrade the myelin fragments.

During the pro-inflammatory phase of WD, Schwann cells initiate the breakdown of the myelin sheath through a process known as myelin autophagy or myelinophagy, a cellular process by which Schwann cells degrade their own intrinsic cellular components (Figure 2). The process begins with the degradation of the myelin into ovoid-shaped segments within activated Schwann cells, followed by the formation of autophagosomes that engulf myelin membranes. After fusing with lysosomes, the autophagosomes proceed through further stages of classical phagocytosis. Schwann cells first fragment the myelin sheath into intracellular, ovoid-shaped segments near the Schmidt–Lanterman incisures. These myelin ovoids gradually break down into smaller intra- and extra-cellular debris along the distal segment of the injured axon [79,80,81]. Myelinophagy is regulated by autophagy-related genes (*Atgs*), particularly *Atg7* in Schwann cells, as well as by the JNK/c-JUN pathway, a central regulator of Schwann cell-mediated repair processes [79].

Beyond the initial role of Schwann cells, the infiltration of neutrophils and monocytes at the injury site, together with nerve-resident endoneurial macrophages, is required for early myelin clearance [8,82,83]. Thus, the site of nerve injury involves coordinated interactions between Schwann cells and distinct immune cell types, not only for the clearance of cellular debris but also for facilitating nerve repair. Immune cell recruitment during the early phase of WD is initiated by a pro-inflammatory response of Schwann cells to damage-associated molecular patterns (DAMPs) through Toll-like receptors (TLRs) [84,85]. This process is accompanied by the coordinated upregulation and release of cytokines and chemokines [2,3,65,86].

Neutrophils are among the first immune cells recruited to the site of nerve injury, and, along with Schwann cells, they release cytokines such as tumor necrosis factor-α (TNF-α), interleukin 1β (IL-1β), and interleukin 6 (IL-6), as well as a broad spectrum of chemokines [8,87]. They contribute to the clearance of cellular debris; however, they also release neutrophil extracellular traps, which impede nerve regeneration by inhibiting macrophage infiltration. Owing to their short lifespan and rapid apoptosis, this detrimental effect is restricted to the first days after nerve injury [88]. Moreover, granules released during neutrophil apoptosis can promote macrophage recruitment and activation [89].

The inflammatory profiling of Schwann cells is characterized by the release of cytokines and chemokines that promote the recruitment of blood-borne macrophages into the endoneurium distal to the site of nerve injury [2,9,86,90]. Distinct macrophage subsets, defined by specific molecular markers, populate different compartments of the peripheral nervous system at particular time points following nerve injury, likely determining their beneficial or detrimental roles [91,92,93]. Macrophages are classically categorized into pro-inflammatory and anti-inflammatory phenotypes [9,94]. Pro-inflammatory M1 macrophages, derived from infiltrating monocytes, predominate during the early phase of nerve injury, where they mediate the phagocytosis of myelin and cellular debris. Subsequently, these M1 macrophages are replaced by anti-inflammatory M2 macrophages, whose subtypes attenuate the inflammatory response and promote nerve regeneration. It is now well established that there is no sharp boundary between the M1 and M2 macrophage subpopulations; instead, macrophage polarization represents a continuum of activation states, and injury-associated macrophages exhibit dynamic changes in their transcriptional profiles [9,95].

The classic division of macrophages into M1 and M2 subpopulations has been further refined by molecular markers, single-cell analyses, and spatial transcriptomic analyses, revealing a far greater degree of macrophage heterogeneity [67,93,95,96]. Single-cell RNA-seq analysis of intact mouse sciatic nerves identified two major subsets of macrophages residing in distinct nerve compartments. Relmα^−^Mgl1^−^ macrophages resided in the endoneurium, whereas Relmα^+^Mgl1^+^ macrophages were found in the epineurium of the sciatic nerve. These two resident macrophage populations respond differently to nerve injury. Epineurial Relmα^+^ Mgl1^+^ resident macrophages do not respond to injury signals, whereas endoneurial Relmα^−^Mgl1^−^ macrophages become transiently activated and segregate into two subgroups with distinct gene expression profiles. A population of *S100a4*-expressing macrophages and a population of *H2-Aa*-expressing macrophages are considered to represent newly recruited monocyte-derived macrophages associated with the wound microenvironment [93]. In addition, single-cell transcriptomic analyses have also revealed the temporal transition of five macrophage clusters characterized by distinct gene expression profiles. Macrophage subtype 0, detected one day after nerve injury, is characterized by metabolic processes associated with acute-phase responses and amino acid metabolism. By day 3, subtype 0 macrophages are largely replaced by subtype 1 macrophages, which represent a substantial fraction of blood-derived macrophages primarily involved in cytokine regulation. Subtype 2 macrophages, characterized by the expression of *Dcn*, *Col3a1*, and *Col1a1*, are functionally associated with extracellular matrix remodeling. By days 5 and 7, the numbers of subtype 1 and subtype 2 macrophages decline, coinciding with a progressive increase in subtype 3 and 4 macrophages. Subtype 3 macrophages contribute to the regulation of hematopoiesis, whereas subtype 4 macrophages are involved in epigenetic regulation, particularly through pathways associated with the negative regulation of cGAS/STING signaling [67].

The dynamic and tightly regulated inflammatory profiling of both Schwann cells and macrophages creates a pro-inflammatory environment that ensures the clearance of axonal and myelin debris distal to the nerve lesion, as well as promoting nerve wound healing at the gap between the stumps of complete interruption of nerve continuity. Schwann cells migrate from both the proximal and distal stumps into the nerve gap [97], where they form cellular cords that guide regenerated axons across the gap toward the distal stump. Interestingly, this is preceded by hypoxia within the nerve bridge, which stimulates macrophages to promote the formation of a polarized vasculature that directs Schwann cell migration [98,99]. Transforming growth factor-β (TGF-β) signaling promotes Schwann cell migration by facilitating crosstalk with Eph signaling through N-cadherin. In addition, the local TGF-β within the nerve gap induces the emergence of a distinct Schwann cell subpopulation with enhanced mesenchymal characteristics, thereby promoting bridge formation and subsequent axonal regeneration [100,101].

In summary, a key response of myelinating Schwann cells during the early phase of axonal injury is the induction of myelinophagy, accompanied by their proliferation and dedifferentiation. This initial response is followed by the onset of an inflammatory profile in Schwann cells, associated with the recruitment of neutrophils and macrophages that are involved in the clearance of myelin and axonal debris. Subsequently, pro-inflammatory Schwann cells and macrophages are replaced by regeneration-supporting subtypes. These cellular events, as a manifestation of the innate immune response to nerve injury, play a critical role in nerve repair.

### 3.3. Molecular Mechanisms Underlying Schwann Cell Responses to Axonal Injury

Although structural disintegration and physiological manifestations are not immediately apparent during the early phase of axonal injury [102], ongoing molecular changes may trigger Schwann cell responses. Experimental evidence indicates the parallel activation of multiple signaling pathways, which can interact to coordinate these early cellular events, ultimately leading to the establishment of repair Schwann cells.

Given the robust increase in and key role of SARM1 in the final stages of complete axonal degeneration, it has been suggested that SARM1 may also contribute to the early response of Schwann cells and their transformation into a repair phenotype. Recent experimental findings support this possibility, indicating that SARM1 may be involved in the Schwann cell repair response following peripheral nerve injury [56,103]. Although immunohistochemical analysis has not detected the SARM1 protein in Schwann cells, a more sensitive method has identified *Sarm1* mRNA in these cells [104,105]. Further support for a role of SARM1 in early Schwann cell responses comes from the absence of a significant increase in JNK phosphorylation in Schwann cells distal to sciatic nerve transection in *Sarm1^−/−^* mice [106]. JNK is a major MAPK that regulates c-JUN, a key transcription factor driving the Schwann cell repair program [107,108].

Neuregulin 1 (NRG1), acting via the receptor tyrosine kinase ErbB2, provides additional signaling involved in both the dedifferentiation and redifferentiation of Schwann cells [109]. Axonal injury induces a rapid and transient activation of ErbB2 in the microvilli of Schwann cells, which are in direct contact with the axons [66]. This activation of ErbB2 is associated with an increased expression of c-JUN and NOTCH, both of which are negative transcriptional regulators of myelination and promote Schwann cell transdifferentiation [110]. Rapid and robust activation of extracellular signal-regulated kinase (ERK) signaling in Schwann cells following nerve injury represents another key pathway, contributing to the downregulation of myelin-specific genes and the upregulation of genes associated with the dedifferentiated state [108,111]. Interestingly, ERK activation in Schwann cells can also be triggered via TLR pathways [112] in response to DAMPs [85].

Cellular and molecular reactions occur following nerve injury, similar to injuries in other peripheral tissues, to establish conditions favorable for regeneration. In the early stages of this processes, cell death leads to the release of various DAMPs, which activate the innate immune response. This innate immune activation is widely considered to be essential for the initiation and progression of tissue regeneration [113]. Damaged axons and their surrounding myelin sheaths constitute a significant source of various DAMPs, including heat shock proteins (HSPs), S100 proteins, MBP, galactocerebrosides (Gal-C), and oxidized lipids. These molecules, released during the early phases of WD, contribute to the induction of the repair phenotype in Schwann cells [114,115,116]. The released DAMPs activate TLRs and other pattern-recognition receptors (PRRs), triggering innate immune reactions distal to the injured nerve. These responses are characterized by the recruitment of macrophages and other immune cells, as well as by the inflammatory reprogramming of repair Schwann cells [2,84,117,118].

As noted above, peripheral axons contain numerous mitochondria, which accumulate in sensory and motor axons at their peripheral terminals, nodes of Ranvier, and Schmidt–Lanterman cleft regions where Schwann cells are directly affected. In injured axons, mitochondria become a significant source of mitochondrial DAMPs (mtDAMPs), including formylated peptides and mtDNA, which trigger innate and inflammatory responses following nerve injury [119,120]. The release of mtDAMPs is facilitated by mPTPs, which are activated by early increases in intracellular calcium levels in injured axons [20,25]. Subsequently, the released mtDAMPs induce early inflammatory reprogramming of Schwann cells via formyl peptide receptor 2 (FPR2) and Toll-like receptor 9 (TLR9) [85,121,122].

Due to phenotypic changes occurring in early response to axonal injury, activated Schwann cells acquire an inflammatory profile [2,123], which enables them to recruit neutrophils and macrophages that continue the clearance of myelin debris during the early phase of WD [2,118]. In addition, the early upregulation of certain cytokines in Schwann cells via c-JUN is associated with the induction of their repair status [124]. In later stages of WD, cytokines and chemokines, together with neurotrophins released by repair Schwann cells, contribute to the promotion of axon regrowth and regeneration (see below).

## 4. Wallerian Degeneration Generates Extrinsic Conditions to Support Axon Regeneration

A critical determinant of successful axon regeneration is the timely transition from a pro-inflammatory to a pro-regenerative microenvironment during WD. Thereafter, the resolution of inflammatory profiling of Schwann cells and macrophages and their transition into pro-regeneration stages, together with the recruitment of additional immune cells, the activation of endoneurial fibroblasts, and the remodeling of the endoneurial ECM, defines the pro-regenerative phase of WD distal to the site of nerve injury [67,125]. This pro-regenerative phase establishes a permissive environment for axon regeneration.

The transition from a pro-inflammatory to a pro-regenerative environment is orchestrated primarily by TGF-β, a multifunctional cytokine that acts as a master regulator of the tissue repair response, mainly through the canonical Smad2/3-Smad4 and the non-canonical AKT signaling pathways [126]. TGF-β signaling regulates Schwann cell reprogramming, the phenotypic transition and differentiation of fibroblast and macrophage subsets, and the modulation of endothelial and infiltrating immune cells, thereby coordinating multiple cellular processes essential for peripheral nerve regeneration [67,126,127,128].

### 4.1. Repair Schwann Cells

Schwann cells distal to the site of nerve injury initially undergo reprogramming to a pro-inflammatory phenotype, followed by a transition to repair Schwann cells, a specialized phenotype that promotes axonal regeneration. Although the molecular changes underlying the transition from myelin-forming to repair Schwann cells have been well characterized, accumulating evidence indicates that repair Schwann cells can also arise from non-myelinating (Remak) Schwann cells [129]. However, the specific phenotypic changes involved in this transition of non-myelinating Schwann cells remain poorly understood.

In the context of Schwann cell reprogramming following axonal injury, it is important to emphasize that, during development, immature Schwann cells are trophically dependent on axons, which also direct their differentiation [130]. However, Schwann cells that survive the loss of axonal contact after nerve injury and undergo reprogramming to a repair state represent a fundamentally different biological scenario, reflecting an adaptive reprogramming process rather than a recapitulation of developmental events [5,107,131,132]. Therefore, Schwann cell activation and reprogramming after nerve injury, together with their role in axon regeneration, should not be viewed as a mere recapitulation of ontogenetic development. Furthermore, because mature Schwann cells not only downregulate genes associated with their differentiated phenotype but also activate repair-associated gene programs, their transition to the repair phenotype can be regarded as a form of transdifferentiation [132].

Dynamic transcriptional regulation plays an essential role in the molecular mechanisms governing Schwann cell transdifferentiation into the repair phenotype. During WD distal to the site of nerve injury, TFs coordinately down- or up-regulate gene expression programs that define successive stages of Schwann cell phenotypic transition. The initial phase of Schwann cell reprogramming is characterized by the rapid silencing of the transcriptional network that maintains myelin homeostasis. This process is driven primarily by the suppression of Egr2 (Krox20) and octamer-binding factor 6 (Oct6), the master regulators of peripheral myelination, together with their downstream signaling pathways. Consequently, the synthesis of major myelin proteins, including P0 (MPZ), myelin basic protein (MBP), myelin-associated glycoprotein (MAG), peripheral myelin protein 22 (PMP22), and periaxin, is markedly reduced. The transition to the repair Schwann cell phenotype is subsequently accompanied by the upregulation of TFs, including c-Jun, Sox-2, Pax-3, and Notch, which induce the synthesis of regeneration-associated molecules such as Sonic Hedgehog (SHH), glial cell line-derived neurotrophic factor (GDNF), and Artemin [133,134]. c-Jun is required for Schwann cell dedifferentiation and the maintenance of their repair phenotype [107], in part by inhibiting myelination through the suppression of Krox-20, a transcription factor that activates myelin gene expression [35,135,136].

Plasticity and maintenance of the repair Schwann cell phenotype are critical for successful peripheral nerve regeneration [131]. The transcription factor c-Jun, a master regulator of Schwann cell plasticity after peripheral nerve injury, is regulated by an integrated network of MAPK signaling pathways, including JNK, ERK1/2, and p38 MAPK. These pathways are rapidly activated after peripheral nerve injury and converge on the AP-1 transcriptional complex, of which c-Jun is a principal component. Among these pathways, JNK is the primary kinase responsible for phosphorylation and activation of c-Jun, thereby enhancing AP-1-dependent transcription and promoting Schwann cell dedifferentiation, acquisition of the repair phenotype, and expression of regeneration-associated genes [107,132]. ERK1/2 signaling is also rapidly activated in Schwann cells both at the site of nerve injury and throughout the distal nerve stump. This suggests that ERK1/2 signaling cooperates with c-Jun to regulate Schwann cell phenotype reprogramming during WD. Sustained activation of ERK1/2 suppresses the myelinating program by inducing c-Jun expression and antagonizing the activity of the myelination-associated transcription factor Krox20/Egr2. This coordinated ERK1/2–c-Jun signaling axis enables mature Schwann cells to undergo reversible phenotypic reprogramming while preserving their capacity for subsequent remyelination [108,132].

The p38 MAPK pathway provides an additional regulatory mechanism that links injury-induced pro-inflammatory signaling with Schwann cell plasticity. Activation of p38 promotes the induction of inflammatory and stress-response genes, regulates cytokine production, and facilitates efficient myelin clearance [65,118,132]. Although p38 does not directly activate c-Jun to the same extent as JNK, it cooperates with other MAPK pathways to sustain the transcriptional program associated with repair Schwann cells. In addition, p38 MAPK acts as a negative regulator of Schwann cell differentiation and myelination, thereby contributing to the maintenance of the repair phenotype following peripheral nerve injury [11,108,132].

Collectively, the ERK1/2, JNK, and p38 MAPK pathways constitute an integrated signaling network that converges on c-Jun to regulate the balance between Schwann cell plasticity and differentiation. The magnitude, duration, and temporal coordination of MAPK activation determine whether Schwann cells maintain the pro-regenerative repair phenotype or revert to a differentiated myelinating state. Dysregulation of this regulatory network may contribute to impaired regeneration in chronic denervation and inflammatory neuropathies, highlighting these pathways as attractive therapeutic targets for restoring Schwann cell regenerative capacity.

Schwann cells that have lost axonal contact display an early wave of IL-1β mRNA upregulation and IL-1β protein secretion within hours [2]. IL-1β activates JNK and ERK pathways, leading to increased c-JUN expression in Schwann cells. This early activation of c-JUN by IL-1β plays a central role in reprogramming Schwann cells into the repair phenotype [107,124,137]. In addition to IL-1β, NRG1/ErbB2 signaling also contributes to elevated c-JUN levels in Schwann cells [109,110]. As discussed above, the delayed appearance of c-JUN-positive repair Schwann cells in Sarm1^−/−^ mice further supports the notion that signaling from degenerating axons plays a direct role in Schwann cell phenotypic reprogramming [56].

The early phase of Schwann cell reprogramming is also characterized by the upregulation of the glial fibrillary acidic protein (GFAP), neural cell adhesion molecule (NCAM), and p75 neurotrophin receptor (p75NTR), molecular markers of immature Schwann cells that resemble those expressed during peripheral nerve development [135,138]. Finally, repair Schwann cells upregulate numerous axon growth-promoting molecules, including neurotrophic factors, neuropoietic cytokines and their receptors, and cell adhesion molecules, thereby creating a microenvironment that supports axon regeneration [2,139,140,141,142].

Repair Schwann cells, aligned to form the bands of Büngner, promote axon regeneration through the release of neurotrophic molecules and exosomes, followed by direct interactions between regenerating growth cones and either repair Schwann cells or the endoneurial ECM via adhesion molecules (see below).

### 4.2. Neurotrophic Factors and Exosomes

Repair Schwann cells are a major source of neurotrophins, including nerve growth factor (NGF), brain-derived neurotrophic factor (BDNF), neurotrophin-3 (NT-3), and neurotrophin-4/5 (NT-4/5) (for a review, see [140]), as well as other types of neurotrophic factors, such as vascular endothelial growth factor (VEGF), hepatocyte growth factor (HGF), and insulin-like growth factor 1 (IGF-1). The biological effects of neurotrophins are mediated through the low-affinity receptor p75NTR and high-affinity neurotrophin receptors. The latter consists of the protein tyrosine kinase receptors TrkA, TrkB, and TrkC, which bind their ligands with higher specificity [143]. Experimental studies show that neurotrophic factors play a significant role in promoting regenerative axon growth not individually, but synergistically [144]. The axon growth-promoting effects of neurotrophins are further enhanced by their parallel action with some cytokines. Thus, the inflammatory profile of repair Schwann cells not only supports the production of neurotrophic factors but also potentiates their ability to promote axon regeneration [142,145].

Although experimental evidence supports the beneficial role of inflammatory mediators in nerve repair and axon regeneration [65,142,146,147,148], it is important to emphasize that excessive or prolonged inflammation in the damaged nerve can delay or impair axon regeneration. Therefore, maintaining physiological levels of inflammatory mediators is essential for the successful functional reinnervation of target tissues and represents a major challenge for future experimental and clinical research.

In addition to direct secretion of neurotrophic factors, repair Schwann cells generated during WD may facilitate axonal regeneration through intercellular communication involving extracellular vesicles (EVs), particularly exosomes. These exosomes are small, membrane-bound vesicles enriched with regenerative microRNAs (miRNAs), cytokines, and other molecules that promote axonal outgrowth, modulate inflammatory responses, and support neuronal survival [149,150,151]. It was evidenced that Schwann cell exosomes can be internalized by regenerated axons to support their outgrowth [152,153]. In addition, Schwann cell-derived exosomes exert anti-inflammatory and immunoregulatory effects by reducing the polarization of pro-inflammatory macrophages and the release of inflammatory cytokines [154]. Together, these findings underscore the importance of exosome-mediated communication as a supplementary yet potent pathway through which repair Schwann cells support peripheral nerve regeneration. In contrast to cell therapy, small exosomes may readily penetrate target cells in injured nerves and improve axon regeneration, including repair of severe nerve damage [155,156,157].

### 4.3. The Endoneurial ECM Changes

The endoneurial ECM molecules of mammalian peripheral nerves play a crucial role in the arrangement of the appropriate extrinsic environment to maintain functional nerve integrity. It is composed of a variety of structural proteins (e.g., collagen types I, III, and IV); glycoproteins such as laminins, fibronectin (FN), tenascin-C (TN-C), and thrombospondins (TSPs); and proteoglycans and glycosaminoglycans, including heparan sulfate proteoglycans and chondroitin sulfate proteoglycans (CSPGs) [158].

Remodeling of the endoneurial ECM during WD is mediated by matrix metalloproteinases (MMPs) and other endopeptidases released by Schwann cells and infiltrating immune cells. Following nerve injury, MMPs, particularly MMP-2 and MMP-9, are significantly upregulated. MMP-9 is rapidly expressed at the injury site, where it is associated with the breakdown of the blood–nerve barrier and the recruitment of macrophages. MMP-2, which is constitutively expressed by Schwann cells, is upregulated and activated several days post-injury, contributing to the endoneurial ECM remodeling during WD [159]. A subset of endoneurial neurofibroblasts also plays a significant role in modulating the endoneurial ECM during the early phase of WD [67].

The basal lamina of Schwann cells is a specialized component of the endoneurium responsible for the growth and correct guidance of regenerated axons. Laminin heterotrimers, collagen IV, nidogen or entactin, and proteoglycans (e.g., perlecan and agrin) are typical molecular components of the basal lamina produced by Schwann cells during their maturation [160]. The altered molecular composition of the endoneurial ECM, including basal lamina tubes, can exert not only facilitatory [158,161,162,163,164] but also inhibitory effects on axonal regeneration. For example, CSPGs are upregulated distal to a nerve injury and inhibit axon growth. However, when CSPGs are cleaved from the basal lamina by activated MMPs, the extracellular environment becomes more permissive to subsequent axonal regeneration [165,166,167].

Modulation of the endoneurial ECM distal to the site of nerve injury facilitates cellular migration and proliferation during the early phase and promotes axonal regrowth during the later stages of WD [168,169]. Integrins, which constitute a major family of ECM receptors, mediate these interactions and transduce ECM-derived signals into intracellular responses [170,171]. Integrin signaling from the endoneurial ECM regulates Schwann cell morphology during migration and maturation, including radial sorting and the subsequent myelination of regenerated axons [172]. In addition, integrins mediate crosstalk between the endoneurial ECM and intracellular signaling pathways that promote axonal regeneration [173,174]. For example, α9 integrin supports axonal growth on tenascin-C-containing substrates [175,176].

The endoneurial ECM, which undergoes dynamic remodeling during WD, serves not only as a structural scaffold for growth cones of regenerating axons but also as a local reservoir for neurotrophic factors and cytokines, thereby regulating their bioavailability. Although neurotrophins and cytokines are highly diffusible molecules, their binding to the endoneurial ECM restricts their diffusion and enables the formation of localized high-concentration depots that enhance signaling to adjacent cells and promote axon growth [159,169,177,178]. Consequently, a detailed understanding of the endoneurial ECM composition and its interactions with neurotrophic factors and cytokines remains a critical challenge for advancing axon regeneration strategies, particularly in the development of engineered nerve conduits [169].

### 4.4. Extrinsic Conditions Distal to Nerve Injury That Support Specific Axon Regeneration

An axonal lesion with preserved continuity of the endoneurial ECM is called axonotmesis, whereas in the most severe form of injury, neurotmesis, both the axons and the endoneurial connective tissue are completely disrupted. In the latter case, accurate navigation of regenerating sensory and motor axons to their appropriate target tissues is critical for successful functional reinnervation [179].

It has been demonstrated that Schwann cells and their endoneurial ECM become specialized during differentiation for motor or sensory axons. Denervated motor and sensory Schwann cells exhibit the expression of distinct patterns of neurotrophic factors [16,180,181]. Moreover, the repair Schwann cells of axotomized motor nerve fibers retain their motor axon phenotype, even in the presence of neighboring sensory fibers in mixed nerves [180,182]. Similarly, differences in the molecular composition of the endoneurial ECM associated with motor and sensory axons have been identified [17,183]. Therefore, the specific molecular composition of the endoneurial ECM, together with the distinct patterns of neurotrophic factors released by repair Schwann cells that retain their motor axon phenotype following WD, contributes to preferential axonal navigation. The process known in experimental models as preferential motor reinnervation depends on a 3-week course of WD to optimize specific conditions for motor axon regeneration [180,182]. However, other experimental results and clinical observations suggest that differences in axon-specific phenotypes of Schwann cells and the endoneurial ECM do not play a critical role in promoting axon regeneration after saphenous or sural nerve grafting of a mixed nerve. These differences may only delay axon regeneration and maturation [184,185].

**Figure 3:**
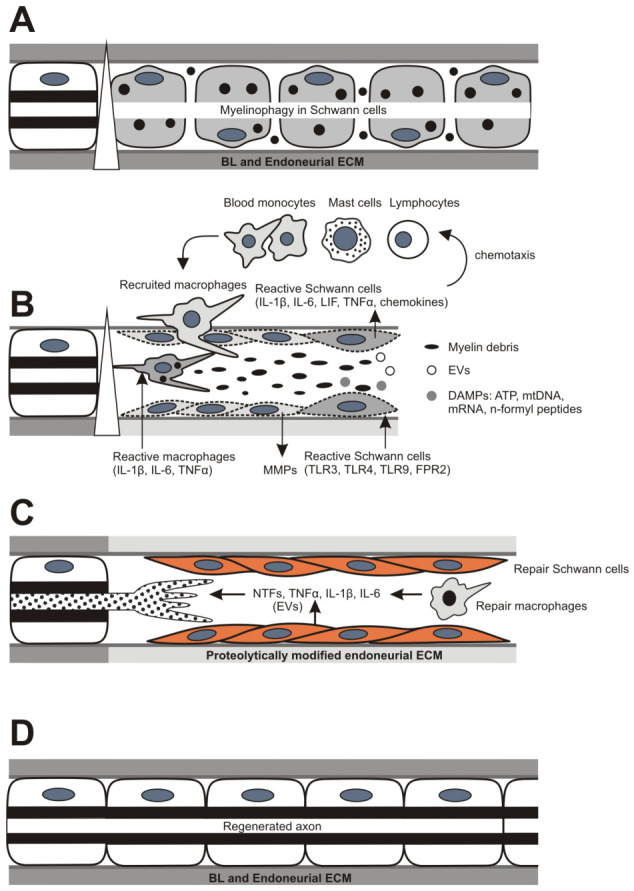
This diagram illustrates the basic cellular and molecular events that occur following axonal injury in the peripheral nerve. (**A**) Early phase after injury. Myelin sheath debris is initially cleared by Schwann cells through myelinophagy. (**B**) Pro-inflammatory phase of Wallerian degeneration. Schwann cells proliferate, dedifferentiate, and are reactivated by DAMPs via PRRs. Reactive Schwann cells release MMPs to modify the endoneurial ECM, and they secrete cytokines and chemokines to recruit immune cells, including macrophages, which assist in myelin debris clearance. (**C**) Pro-regenerative phase of Wallerian degeneration. Repair Schwann cells, aligned into bands of Büngner, together with macrophages, undergo a phenotypic transition from a pro-inflammatory to a pro-regenerative state. They release neurotrophic factors, cytokines, chemokines, and EVs, thereby creating a permissive microenvironment that supports axon regeneration. (**D**) Final stage distal to a nerve injury showing successful regeneration of the injured axon and reinnervation of the distal nerve segment.

The basic cellular and molecular events occurring distal to axonal injury in the peripheral nerve are summarized in the diagram shown in Figure 3.

## 5. Wallerian Degeneration as a Source of Molecular Signaling to Induce Intrinsic Regeneration Program of Axotomized Neurons

Metabolic and molecular changes are triggered in axotomized neuronal cell bodies. A critical requirement for axon regeneration following nerve injury is the survival of injured neurons and the activation of their intrinsic regeneration program. Both processes are regulated and coordinated by specific families of regeneration-associated genes (RAGs), which encode molecules across several categories, including cytoskeletal proteins and adaptors, metabolic enzymes, neuropeptides, cytokines and chemokines, neurotrophins, and transcription factors [186]. The expression of RAGs is under the control of various transcriptional regulatory mechanisms [187]. Axonal injury initiates both intrinsic, neuron-autonomous transcriptional reprogramming and extrinsic signaling mechanisms mediated by injury molecular signals transported retrogradely along the axon.

### 5.1. Neuron-Autonomous Transcriptional Reprogramming

Neuron-autonomous transcriptional reprogramming is driven by injury-responsive transcription factors (TFs), whose expression and activity are directly regulated in neurons early after axonal injury. The initial rise in calcium levels during WD triggers a cascade of subsequent reactions, inducing a calcium wave that propagates to the neuronal cell body via the action of L-type voltage-gated calcium channels [188]. This injury-induced, back-propagating calcium wave activates protein kinase C (PKC), which promotes the nuclear export of histone deacetylase 5 (HDAC5), thereby increasing histone acetylation and inducing the expression of RAGs [187,189]. Histone acetylation and demethylation represent the final regulatory steps in RAG expression, mediated by TFs upregulated in axotomized neurons [187]. The increased level of intraneuronal calcium induced by WD is also involved in an additional mechanism regulating TFs. This is associated with elevated cAMP levels, which affect various injury-associated proteins, including cAMP-responsive element-binding protein (CREB) and other pro-regenerative TFs in parallel [10,190].

Activating transcription factor 3 (ATF3), c-JUN, and SMAD family member 1 (SMAD1), as well as STAT3 activated by phosphorylation, are among the most frequently studied TFs that are upregulated in axotomized and regenerating neurons [191,192,193,194]. Several lines of evidence also suggest that TFs may cooperate within a large network of signaling pathways that drive the transcriptional regulation of RAGs. One crucial cooperative interaction is between c-JUN and ATF3, which are upregulated within hours after axonal injury and act synergistically to enhance the expression of RAGs (e.g., *GAP43* and *SPRR1A*), thereby promoting neuronal survival and axonal outgrowth [189].

### 5.2. Extrinsic Signaling Mechanisms

Given the distance between neuronal bodies and the site of axonal injury, retrograde axonal transport of injury signals is required to sustain the regulation of RAGs. Various injury signals locally activated during WD have been shown to undergo retrograde transport along axons via the importin–dynein complex, enabling them to contribute to the regulation of the neuronal intrinsic regeneration program.

One example of a locally activated and retrogradely transported transforming factor is STAT3. It is activated by phosphorylation in response to the cytokines released during WD after nerve injury. Once phosphorylated, STAT3 is retrogradely transported via dynein and importin α5, and subsequently translocated to the nucleus, where it modulates the survival of axotomized neurons [195]. In addition to promoting neuronal survival, nuclear STAT3 enhances the transcription of RAGs, thereby facilitating axonal regeneration [196]. Interestingly, STAT3 activation and nuclear translocation induced by IL-6, which promotes the intrinsic regeneration program, can also occur in neurons not directly associated with the injured nerve. This finding suggests the presence of alternative pathways for transmitting injury signals from the damaged nerve to neuronal bodies [197,198].

MAPKs represent another family of retrogradely transported injury signals that activate TFs in axotomized neurons [10,12]. Following peripheral axotomy, ERK is phosphorylated and forms a complex with vimentin and importin-β, enabling its retrograde transport and contributing to the initiation of the neuronal regeneration program. However, while phosphorylated ERK is involved in initiating regeneration, it is not required for subsequent axonal outgrowth [199]. JNK, which is essential for the transition of myelinating Schwann cells into a repair phenotype [200], also plays a pivotal role in retrograde injury signaling to the neuronal cell body, where it is necessary for the activation of TFs such as c-JUN and ATF3 in the neuronal nucleus [201].

DLK-1 is an important neuronal stress response kinase that functions as a master regulator of multiple signaling pathways involved in both degeneration and regeneration. Loss of cytoskeletal integrity activates DLK-1 in both the proximal and distal segments of the injured axon. As described in Section 2.1, the DLK-1/JNK signaling pathway plays a key role in initiating the axon degeneration program through its interaction with the SARM1 pathway [18,57]. Conversely, this pathway is also critical for the transcriptional responses of neurons following axonal injury [202,203,204]. In the proximal segment, the DLK-1/JNK signaling complex is retrogradely transported to the neuronal soma, where it activates c-JUN, leading to the upregulation of genes that promote axon regeneration [189,205]. This dual function of the DLK-1/JNK signaling pathway appears to depend on the neuronal compartment in which signaling occurs (distal axon stump vs. neuronal soma), as well as the time period of activation during which gene expression is regulated.

## 6. Impaired Regenerative Capacity of Schwann Cells in Chronically Denervated Peripheral Nerves

### 6.1. Regenerative Capacity of Repair Schwann Cells in Chronically Denervated Distal Nerve Stumps

The regenerative competence of repairing Schwann cells is not maintained indefinitely. Experimental models of chronic denervation have consistently demonstrated that prolonged absence of axonal contact results in progressive deterioration of the repair phenotype. Within weeks to months, chronically denervated Schwann cells exhibit a reduced proliferative capacity, diminished expression of regeneration-associated genes, impaired secretion of neurotrophic factors, and partial loss of the aligned cellular architecture characteristic of bands of Büngner. These changes are accompanied by increasing fibrosis, endoneurial ECM remodeling, adipocyte infiltration, and reduced vascular support, all of which create an increasingly hostile environment for subsequent axonal regeneration [206,207]. Chronic denervation also reduces the expression of Schwann cell-specific molecular markers [208] and axon-promoting genes, including Gdnf, Bdnf, Ntf3, and Ngfr, resulting in a significant reduction in the ability of the distal nerve stumps to support axon regeneration [209].

The repair phenotype of Schwann cells is not stable; it undergoes a progressive functional decline, structural atrophy, and cellular senescence. The long-term denervated Schwann cells retain their repair phenotype through autocrine signaling loops involving, for example, Shh expression, which is strongly upregulated in repair Schwann cells after injury [210] and activated signal transducer and activator of transcription 3 (STAT3) [211]. With prolonged chronic denervation, Shh expression gradually declines [210,212], whereas activated STAT3 is maintained throughout long-term denervation [211]. In addition, reduced expression of c-Jun, which acts together with Sox2, Pax3, and Notch to maintain the highly plastic repair Schwann cell phenotype, results in impaired axon regeneration [208,210,213]. Diminished c-Jun expression in the Schwann cells of a chronic denervated nerve stump is associated with their transition into a senescent phenotype that inhibits axonal regeneration [214].

Both experimental and clinical evidence indicates that the regenerative capacity of repair Schwann cells declines during long-term denervation [208,212]. This highlights the need for therapeutic strategies to preserve the repair phenotype and regeneration capacity of Schwann cells in chronically denervated peripheral nerves. Recently, cell-based therapies and pharmaceutical interventions have emerged as promising approaches [215].

Axon regeneration through chronically denervated nerve stumps is enhanced by the administration of mesenchymal stem cells [216,217] or skin-derived precursor Schwann cells (SKP-SCs) [218]. Treatment of distal nerve stumps with SKP-SCs increases the number of regenerated axons and improves muscle reinnervation, including the recovery of muscle action potentials [218,219]. Although substantial improvements have been demonstrated in animal models using SKP-SCs, the clinical application of human SKP-SCs requires further investigation before they can be incorporated into clinical practice [220]. Nevertheless, the generation of Schwann cell-like cells from mesenchymal stem cells of various origins represents a promising strategy for promoting axonal regeneration in chronically denervated peripheral nerves, with considerable potential for both preclinical and clinical applications [221,222]. Pharmaceutical interventions include the use of a wide range of neurotrophic factors, growth hormones, and TGF-β1 [215,223].

### 6.2. Wallerian Degeneration and Repair Schwann Cells in Chronic Inflammatory Demyelinating Polyneuropathy

The mechanisms of long-term denervated Schwann cells are highly relevant to chronic inflammatory neuropathies such as chronic inflammatory demyelinating polyneuropathy (CIDP). Although CIDP is primarily considered an immune-mediated demyelinating disease [224,225,226], many patients experience repeated episodes of demyelination, remyelination, conduction block, and secondary axonal degeneration. Persistent inflammation, recurrent immune-mediated injury, and incomplete remyelination expose Schwann cells to prolonged stress that differs fundamentally from the acute injury paradigm. Instead of transient activation followed by successful remyelination, Schwann cells may remain chronically activated or become trapped in an incompletely differentiated state. Over time, repeated cycles of demyelination and denervation may exhaust the plasticity and repair capacity of Schwann cells, leading to progressive impairment of axonal support despite effective immunomodulatory treatment [225,227,228].

In chronic denervation, as also encountered in longstanding CIDP and other peripheral neuropathies, progressive deterioration of the Schwann cell repair phenotype becomes a major limiting factor for successful axonal regeneration. Preservation of Schwann cell plasticity therefore represents a central therapeutic objective that extends beyond neuroimmunomodulation alone. Future interventions combining effective control of inflammation [229,230] with strategies that maintain or rejuvenate repair Schwann cells may substantially improve long-term neurological recovery in chronic peripheral neuropathies. For example, an improved understanding of the underlying immunopathological mechanisms has enabled the development of novel therapeutics that target specific disease-causing pathways. Current approaches focus on B-cell and/or plasma cell depletion using anti-CD20 or anti-CD38 monoclonal antibodies and proteasome inhibitors, IgG depletion through neonatal Fc receptor antagonists, and inhibition of complement activation at various stages of the complement cascade [229]. Moreover, intravenous administration of ultra-high-dose methylcobalamin was evaluated in a small open clinical trial involving patients with immune-mediated or hereditary neuropathy in the chronic progressive or stable phase. The findings indicated that methylcobalamin therapy is both safe and potentially effective in enhancing motor function in chronic peripheral neuropathy, highlighting its promise as a disease-modifying approach to support axonal regeneration in clinical settings [231,232].

A key challenge is harnessing the therapeutic potential of mesenchymal stem cell-derived exosomes to reduce inflammation and rejuvenate senescent Schwann cells in preclinical models of chronically denervated nerves and CIDP, thereby facilitating their future clinical translation.

## 7. Wallerian Degeneration and Axonal Regeneration in Aging Peripheral Nerves

The regenerative potential of mammalian peripheral nerves declines with advancing age [233]. One of the most consistent age-related changes is delayed degeneration and clearance of distal axons and myelin, which is associated with a slowing of WD. The distal nerve stump in aged animals exhibits prolonged persistence of myelin debris, creating an inhibitory environment for regenerating axons and resulting in the accumulation of large myelin debris within macrophages [234]. This delayed removal of myelin debris is attributed to an attenuated immune response involving both Schwann cells and macrophages [235], illustrating that aging is associated with chronic low-grade inflammation that impairs the degeneration-associated phase of WD and its transition to regenerative stages [236,237]. Age-associated defects in lysosomal function and autophagy may further impair myelin degradation.

Moreover, the reduced regenerative capacity of repair Schwann cells, accompanied by the decreased secretion of neurotrophic and neurotropic factors, contributes to impaired axonal regeneration in aging peripheral nerves. This diminished regenerative support provided by aged Schwann cells has been largely attributed to insufficient c-Jun upregulation after injury [210]. Consequently, regenerating neurons receive inadequate trophic support, rendering them more vulnerable to degeneration and neuronal loss.

Furthermore, an age-related decline in neuronal regenerative programs has also been expected. However, several studies suggest that this impairment is primarily due to the age-related alterations in the injured nerve environment and target tissues rather than to a diminished intrinsic growth capacity of neurons or reduced neuronal responsiveness to trophic factors [238].

## 8. Wallerian Degeneration and Advances in Peripheral Nerve Reconstruction

A detailed understanding of the cellular and molecular processes underlying WD has contributed to the development of novel strategies for improving peripheral nerve reconstruction. Peripheral nerve reconstruction is a broad experimental and clinical field [232,239] that is beyond the scope of this review. Nevertheless, several examples illustrate how advances in our understanding of WD have directly influenced approaches to peripheral nerve repair.

Regeneration is particularly successful after nerve compression injuries because the continuity of basal laminae and the endoneurial ECM preserve the original axonal pathways, thereby providing a substrate for accurate axonal regeneration and target innervation [240]. In addition, WD establishes a growth-permissive microenvironment distal to the nerve by removing inhibitory myelin debris and promoting Schwann cell reprogramming, immune cell recruitment, and the endoneurial ECM remodeling.

Peripheral nerve regeneration also occurs after more severe injuries that disrupt nerve continuity and create a gap between the proximal and distal nerve stumps, provided that the defect is appropriately bridged [241]. Fresh autologous nerve grafts remain the clinical gold standard for bridging peripheral nerve defects; however, their use requires harvesting a donor nerve, resulting in donor-site morbidity. Following implantation, fresh nerve grafts undergo WD before becoming permissive for axon regeneration, leading to an initial delay in axonal regrowth. In contrast, predegenerated nerve grafts have already completed WD, allowing Schwann cells and macrophages to remove myelin debris and establish a growth-permissive microenvironment through the secretion of neurotrophic factors. Consequently, the initial delay is reduced, and early axonal regeneration is accelerated [242]. Although predegenerated grafts improve the initial rate of axonal regeneration and preferential motor reinnervation [182], both fresh and predegenerated grafts generally achieve comparable long-term motor and sensory functional recovery in experimental and clinical studies [243].

The development of artificial nerve conduits aimed at replacing autologous nerve grafts is currently progressing rapidly. These conduits are designed to replicate, as closely as possible, the structural and biological properties of the peripheral nerve. Recent progress in nerve conduit design and fabrication has been comprehensively reviewed elsewhere [244]. Importantly, growing knowledge of the cellular and molecular events underlying WD, much of it derived from experimental models, has substantially contributed to the development of next-generation biomimetic nerve conduits. In particular, insights into the composition and regenerative function of the WD-remodeled endoneurial ECM, which provides a highly permissive substrate for axonal regeneration and maturation, together with the incorporation of repair Schwann cells into engineered conduits [245,246], are driving the development of biologically active nerve conduits that more closely recapitulate the regenerative microenvironment of the injured peripheral nerve.

## 9. Conclusions and Perspectives

WD distal to nerve injury involves the intrinsic self-destruction of axons, accompanied by myelin clearance and the transformation of Schwann cells into a repair phenotype. Increased calcium levels distal to the nerve injury, along with retrograde propagation of a calcium wave into neuronal cell bodies, play a pivotal role in triggering downstream molecular responses.

Damaged axons and associated myelin sheaths serve as a significant source of DAMPs, which induce the inflammatory profiling of Schwann cells and stimulate innate immune responses characterized by the upregulation of cytokines and chemokines. These signaling molecules are not only involved in the recruitment of blood-derived immune cells but also act synergistically with neurotrophic factors to facilitate axon regeneration. While the pro-regenerative role of inflammatory mediators is well established, it is crucial to tightly regulate the magnitude and duration of the inflammatory response, as excessive or prolonged inflammation can impair or delay axon regeneration. Given that WD is an innate immune reaction accompanied by neuroinflammation, a key challenge in this field is to determine the spatiotemporal window during which inflammation supports nerve regeneration through beneficial effects on both extrinsic and intrinsic factors, as well as when it subsequently becomes detrimental, leading to secondary nerve damage. Thus, maintaining a balanced inflammatory milieu during WD is critical for effective functional reinnervation of target tissues and for optimizing the performance of artificial nerve conduits.

The endoneurial ECM, which undergoes dynamic remodeling during WD, serves not only as a structural scaffold for the growth cones of regenerating axons but also as a reservoir of neurotrophic factors and cytokines. A deeper understanding of the role of ECM molecules as reservoirs for axon growth-promoting factors is essential for advancing the design of biomimetic nerve conduits capable of replacing autologous nerve grafts, thereby eliminating the donor-site morbidity associated with nerve harvesting.

The generation of repair Schwann cells during WD and the maintenance of their pro-regenerative phenotype are essential for successful peripheral nerve regeneration. Therefore, Schwann cell senescence in chronically denervated nerves and impairment of the repair Schwann cell phenotype in CIDP represents major challenges for both preclinical research and clinical therapy.

Extracellular vesicles (EVs) released by repair Schwann cells and mesenchymal stem cells currently represent a promising therapeutic strategy not only for supporting axonal regeneration but also for reducing the detrimental aspects of WD associated with peripheral neuropathies. Due to their small size, these vesicles can cross barriers within the nervous system and penetrate target cells. Recent studies have demonstrated their ability to enhance axonal regeneration, modulate the inflammatory response during WD, and support neuronal survival and the transition of pro-inflammatory to pro-regenerative phenotypes of Schwann cells and macrophages. The EVs of mesenchymal stem cells or Schwann cell-like cells therefore represent a highly promising therapeutic approach for restoring the repair capacity of senescent Schwann cells and attenuating the detrimental inflammation associated with peripheral neuropathies. Further progress in this field will depend on achieving a more comprehensive characterization of the molecular cargo of EVs derived from distinct cellular sources. Moreover, the critical role of SARM1 in axon degeneration in chemotherapy-induced and other forms of peripheral neuropathy has prompted the development of therapeutic strategies targeting this multidomain protein.

## Data Availability

The original contributions presented in this study are included in the article. Further inquiries can be directed to the corresponding author.
